# Supplementary material for: Infant gut microbiome composition is associated with non-social fear behavior in a pilot study
Source: Nat Commun. 2021 Jun 2;12:3294. doi: 10.1038/s41467-021-23281-y (PMC8172562; doi:10.1038/s41467-021-23281-y)
Supplement: Supplementary file 1 — Supplementary Information [file 41467_2021_23281_MOESM1_ESM.pdf]

SUPPLEMENTARY INFORMATION

Infant Gut Microbiome Composition is Associated with Non-Social Fear Behavior in a Pilot Study

Authors: Alexander L. Carlson[1], Kai Xia[2], M. Andrea Azcarate-Peril[3,4], Samuel P. Rosin[5], Jason, P. Fine[5], Wancen Mu[5], Jared B. Zopp[2], Mary C. Kimmel[2], Martin A. Styner[2,6], Amanda L. Thompson[7,8], Cathi B. Propper[1], Rebecca C. Knickmeyer[2,9,10,11]*

Affiliations:

[1]Frank Porter Graham Child Development Institute, University of North Carolina, Chapel Hill, NC, USA
[2]Department of Psychiatry, University of North Carolina, Chapel Hill, NC, USA
[3]Department of Medicine, University of North Carolina, Chapel Hill, NC, USA
[4]Microbiome Core Facility, University of North Carolina, Chapel Hill, NC, USA
[5]Department of Biostatistics, University of North Carolina, Chapel Hill, NC, USA
[6]Department of Computer Science, University of North Carolina, Chapel Hill, NC, USA
[7]Department of Anthropology, University of North Carolina, Chapel Hill, NC, USA
[8]Department of Nutrition, University of North Carolina, Chapel Hill, NC, USA
[9]Department of Pediatrics and Human Development, Michigan State University, East Lansing, MI, USA
[10]Institute for Quantitative Health Science and Engineering, Michigan State University, East Lansing, MI, USA
[11]C-RAIND Fellow, Michigan State University, East Lansing, MI, USA

*Correspondence to: Rebecca C. Knickmeyer, Institute for Quantitative Health Science and Engineering, Room 2114, Bio Engineering Facility, 775 Woodlot Dr., East Lansing, MI, 48824 USA. Phone: 517-355-3977, Email: knickmey@msu.edu

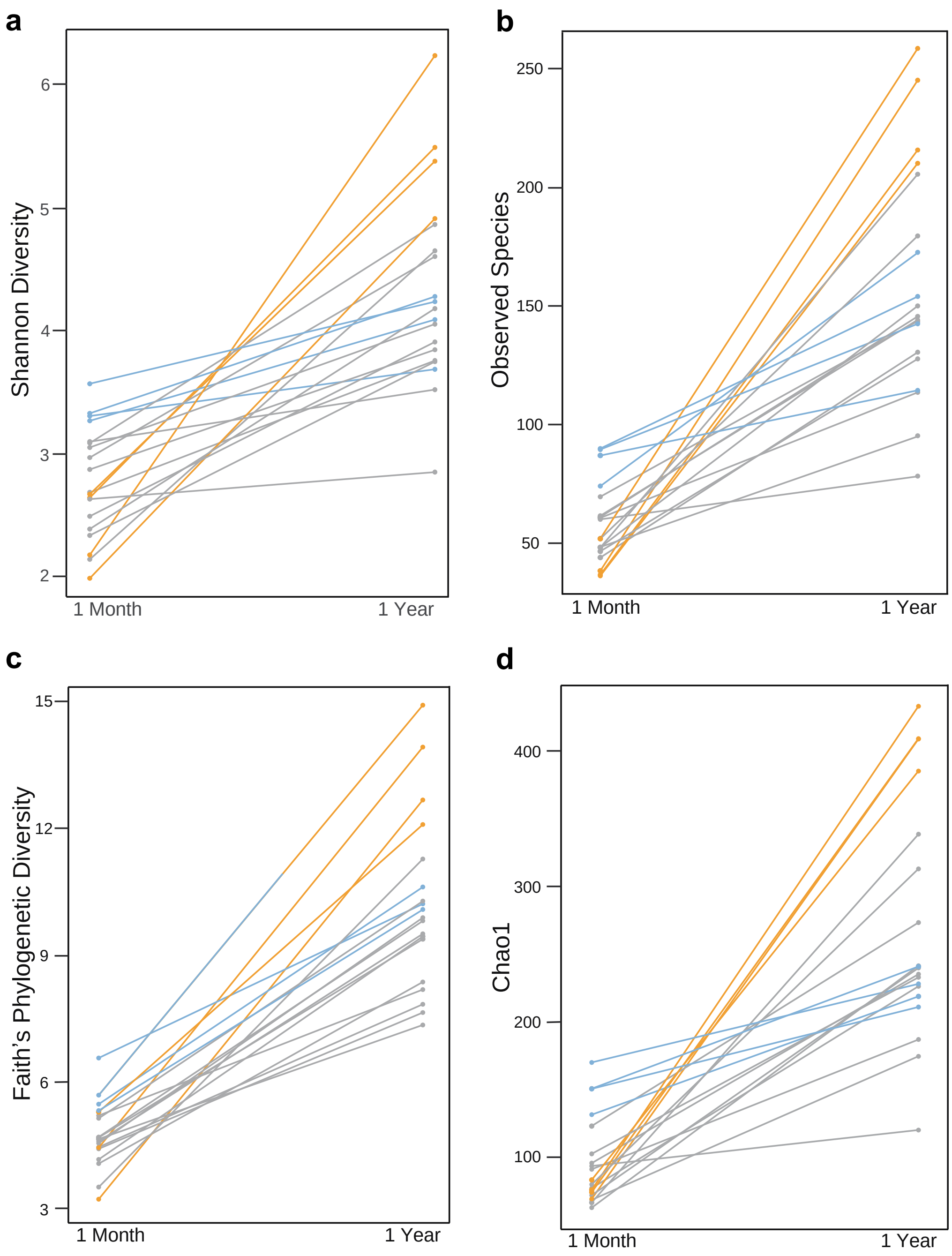

Supplementary Figure 1. Change in alpha diversity from 1 month to 1 year of age. a-d. Orange and blue highlight 4 subjects with highest alpha diversity at 1-year and 1-month, respectively. **a.** Shannon Diversity – Spearman correlation = -0.24 (-.63, .24), n = 19, p = 0.185, estimate = -0.559, std error = 0.405, t = -1.38, CI = (-1.35, 0.23), df = 17, r^2 = 0.10. **b.** Observed Species – Spearman correlation = -0.36 (-0.70, 0.11), n = 19, p = 0.08, estimate = -1.176, Std. Error = 0.635, t = -1.85, CI = (-2.42, 0.07), df = 17, r^2 = 0.17. **c.** Faith's Phylogenetic Diversity – Spearman correlation = 0.17 (-0.31, 0.58), n = 19, p = 0.645, estimate = 0.291, Std. Error = 0.619, t = 0.47, CI = (-0.92, 1.50), df = 17, r^2 = 0.01. **d.** Chao1 – Spearman correlation = -0.52 (-0.79, -0.09), n = 19, p = 0.0559, estimate = -1.279, Std. Error = 0.623, t = -2.05, CI = (-2.50, -0.06), df = 17, r^2 = 0.20. Source data for this figure are provided as a Source Data file.

Supplementary Table 1. Pearson correlations of alpha and beta diversity metrics between 1 month and 1 year of age.

	1-Month Weighted Unifrac PC1	1-Month Weighted Unifrac PC2	1-Month Chao1	1-Month Observed Species	1-Month Faith's Phylogenetic Diversity	1-Month Shannon Diversity	1-Year Weighted Unifrac PC1	1-Year Weighted Unifrac PC2	1-Year Chao1	1-Year Observed Species	1-Year Faith's Phylogenetic Diversity	1-Year Shannon Diversity
1-Month Weighted Unifrac PC1	—	**0.43**	0.13	0.09	0.19	-0.23	-0.35	-0.04	0.14	0.20	0.36	0.25
1-Month Weighted Unifrac PC2	—	—	0.01	0.00	-0.21	-0.23	-0.36	0.18	0.23	0.29	0.35	0.20
1-Month Chao1	—	—	—	**0.95***	**0.55***	**0.39**	-0.03	-0.29	**-0.45**	-0.38	-0.10	-0.22
1-Month Observed Species	—	—	—	—	**0.51***	**0.40**	-0.06	-0.33	**-0.48***	**-0.41**	-0.09	-0.24
1-Month Faith's Phylogenetic Diversity	—	—	—	—	—	0.24	-0.01	**-0.42**	-0.17	-0.06	0.10	0.12
1-Month Shannon Diversity	—	—	—	—	—	—	**0.40**	0.07	**-0.65***	**-0.65***	**-0.47***	-0.32
1-Year Weighted Unifrac PC1	—	—	—	—	—	—	—	-0.08	-0.05	-0.13	-0.08	-0.07
1-Year Weighted Unifrac PC2	—	—	—	—	—	—	—	—	-0.16	-0.24	-0.29	-0.11
1-Year Chao1	—	—	—	—	—	—	—	—	—	**0.97***	**0.85***	**0.84***
1-Year Observed Species	—	—	—	—	—	—	—	—	—	—	**0.88***	**0.87***
1-Year Faith's Phylogenetic Diversity	—	—	—	—	—	—	—	—	—	—	—	**0.85***
1-Year Shannon Diversity	—	—	—	—	—	—	—	—	—	—	—	—

Bold formatting denotes correlations with $p < 0.10$, while * signify correlations with $p < 0.05$. 1-month Weighted Unifrac PC2 and 1-month Weighted Unifrac PC1 ($p = 0.06$), 1-month Observed Species and 1-month Chao1 (p = 7.06E-10), 1-month Faith's Phylogenetic Diversity and 1-month Chao1 ($p = 0.02$), 1-month Faith's Phylogenetic Diversity and 1-month Observed Species ($p = 0.02$), 1-month Shannon Diversity and 1-month Chao1 ($p = 0.0984$), 1-month Shannon Diversity and 1-month Observed Species ($p = 0.09$), 1-year Weighted Unifrac PC1 and 1-month Alpha Diversity ($p = 0.09$), 1-year Weighted Unifrac PC2 and 1-month Faith's Phylogenetic Diversity ($p = 0.08$), 1-year Chao1 and 1-month Chao1 ($p = 0.06$), 1-year Chao1 and 1-month Observed Species ($p = 0.04$), 1-year Chao1 and 1-month Shannon Diversity ($p = 0.003$), 1-year Observed Species and 1-month Observed Species ($p = 0.08$), 1-year Observed Species and 1-month Shannon Diversity ($p = 0.003$), 1-year Observed Species and 1-year Chao1 (p = 1.65E-12), 1-year Faith's Phylogenetic Diversity and 1-month Shannon Diversity ($p = 0.04$), 1-year Faith's Phylogenetic Diversity and 1-year Chao1 (p = 4.93E-06), 1-year Faith's Phylogenetic Diversity and 1-year Observed Species (p = 8.71E-07), 1-year Shannon Diversity and 1-year Chao1 (p = 8.43E-06), 1-year Shannon Diversity and 1-year Observed Species (p = 1.46E-06), 1-year Shannon Diversity and 1-year Faith's Phylogenetic Diversity (p = 4.81E-06). Source data for this table are provided as a Source Data file.

Supplementary Table 2. Genera at 1 year of age associated with non-social fear.

	Facial Fear									Vocal Distress									Bodily Fear								
	q value	*p* value	estimate	std error	*t*	CI 2.5%	CI 97.5%	r^2	df	*q* value	*p* value	estimate	std error	*t*	CI 2.5%	CI 97.5%	r^2	df	*q* value	*p* value	estimate	std error	*t*	CI 2.5%	CI 97.5%	r^2	df
**o__Clostridiales.f__.g__**	0.827	0.119	26.56	16.07	1.65	-4.94	58.06	0.12	15	0.947	**0.045***	30.82	13.92	2.21	3.53	58.11	0.23	14	0.671	0.064	23.90	11.82	2.02	0.73	47.07	0.33	13
Sutterella	0.898	0.308	13.50	12.71	1.06	-11.42	38.41	0.08	13	0.947	0.197	15.87	11.62	1.37	-6.90	38.64	0.16	12	0.671	0.089	16.99	9.18	1.85	-1.00	34.97	0.38	12
Dialister	0.827	0.105	24.81	14.13	1.76	-2.88	52.51	0.18	12	0.947	0.060	26.63	12.73	2.09	1.67	51.59	0.30	11	0.671	0.122	18.71	11.22	1.67	-3.29	40.71	0.36	12
o__Clostridiales.Other.Other	0.827	0.142	33.79	21.57	1.57	-8.48	76.06	0.14	12	0.947	0.135	32.26	20.12	1.60	-7.18	71.70	0.20	12	0.671	0.121	27.85	16.66	1.67	-4.80	60.50	0.33	12
f__Erysipelotrichaceae.g__	0.898	0.377	26.56	28.81	0.92	-29.90	83.03	0.07	11	0.947	0.417	23.24	27.49	0.85	-30.64	77.12	0.09	11	0.992	0.663	10.59	23.62	0.45	-35.71	56.88	0.05	11
Veillonella	0.908	0.605	2.73	5.12	0.53	-7.31	12.76	0.03	11	0.978	0.778	1.42	4.90	0.29	-8.20	11.03	0.01	11	0.992	0.935	-0.35	4.13	-0.08	-8.44	7.75	0.00	11
Bifidobacterium	0.898	0.490	25.08	35.09	0.71	-43.71	93.86	0.05	11	0.947	0.498	23.39	33.32	0.70	-41.91	88.68	0.07	11	0.992	0.515	18.84	28.01	0.67	-36.06	73.75	0.11	11
Collinsella	0.989	0.989	-1.42	97.46	-0.01	-192.44	189.60	0.00	11	0.983	0.983	-2.01	92.42	-0.02	-183.14	179.13	0.00	11	0.992	0.750	-25.23	77.19	-0.33	-176.53	126.06	0.03	11
Bacteroides	0.871	0.238	-1.80	1.44	-1.25	-4.62	1.01	0.12	10	0.947	0.414	-1.20	1.42	-0.85	-3.98	1.57	0.09	10	0.992	0.381	-1.08	1.18	-0.91	-3.40	1.24	0.18	11
Parabacteroides	0.827	0.091	-22.96	12.37	-1.86	-47.20	1.29	0.20	11	0.947	0.169	-18.11	12.27	-1.48	-42.15	5.93	0.21	11	0.671	0.092	-18.21	9.85	-1.85	-37.52	1.10	0.43	11
Prevotella	0.898	0.376	2.58	2.80	0.92	-2.90	8.07	0.07	11	0.947	0.549	1.68	2.71	0.62	-3.64	6.99	0.05	11	0.992	0.967	-0.10	2.32	-0.04	-4.64	4.44	0.00	11
f__Rikenellaceae.g__	0.963	0.684	-28.20	67.41	-0.42	-160.33	103.92	0.02	11	0.947	0.568	-37.35	63.42	-0.59	-161.65	86.95	0.05	11	0.992	0.962	2.61	53.96	0.05	-103.15	108.38	0.00	11
Streptococcus	0.908	0.589	8.50	15.27	0.56	-21.42	38.42	0.03	11	0.947	0.541	9.09	14.41	0.63	-19.16	37.34	0.05	11	0.992	0.831	2.68	12.27	0.22	-21.36	26.73	0.01	11
Clostridium	0.827	0.165	-85.00	56.99	-1.49	-196.69	26.70	0.15	11	0.947	0.128	-87.59	52.91	-1.66	-191.29	16.10	0.25	10	0.781	0.211	-61.30	46.15	-1.33	-151.75	29.14	0.30	11
f__Lachnospiraceae.g__	0.973	0.816	-1.11	4.66	-0.24	-10.24	8.02	0.01	11	0.978	0.733	-1.54	4.40	-0.35	-10.17	7.09	0.02	11	0.992	0.701	-1.45	3.68	-0.39	-8.67	5.77	0.04	11
f__Lachnospiraceae.*g__.Ruminococcus.*	0.973	0.777	-16.18	55.75	-0.29	-125.45	93.08	0.01	11	0.978	0.683	-22.06	52.60	-0.42	-125.15	81.03	0.02	11	0.992	0.366	-40.29	42.83	-0.94	-124.25	43.66	0.19	11
Blautia	0.989	0.981	-3.63	148.16	-0.02	-294.03	286.76	0.00	11	0.982	0.870	-23.45	140.24	-0.17	-298.31	251.41	0.00	11	0.992	0.781	33.37	117.15	0.28	-196.24	262.98	0.02	11
Coprococcus	0.963	0.730	7.71	21.73	0.35	-34.88	50.30	0.01	11	0.978	0.750	6.74	20.62	0.33	-33.68	47.16	0.02	11	0.992	0.910	2.01	17.37	0.12	-32.03	36.05	0.00	11
Dorea	0.885	0.268	73.03	62.41	1.17	-49.30	195.36	0.11	10	0.947	0.351	58.92	60.28	0.98	-59.22	177.07	0.12	10	0.992	0.406	44.15	51.13	0.86	-56.06	144.35	0.17	11
Lachnospira	0.973	0.889	1.05	7.34	0.14	-13.34	15.43	0.00	11	0.982	0.952	0.42	6.97	0.06	-13.23	14.08	0.00	11	0.992	0.992	-0.06	5.84	-0.01	-11.51	11.40	0.00	11
Roseburia	0.827	0.175	26.31	18.11	1.45	-9.18	61.79	0.14	11	0.947	0.341	17.90	17.96	1.00	-17.30	53.11	0.12	11	0.671	0.095	25.20	13.76	1.83	-1.77	52.18	0.42	11
f__Lachnospiraceae.Other	0.898	0.538	-6.98	10.98	-0.64	-28.50	14.55	0.04	11	0.947	0.413	-8.72	10.25	-0.85	-28.82	11.38	0.09	11	0.992	0.728	-3.13	8.78	-0.36	-20.33	14.08	0.03	11
f__Ruminococcaceae.g__	0.963	0.715	1.13	3.01	0.38	-4.78	7.04	0.01	11	0.978	0.830	0.63	2.87	0.22	-5.00	6.26	0.01	11	0.992	0.560	1.43	2.37	0.60	-3.23	6.08	0.09	11
Faecalibacterium	0.898	0.531	2.28	3.52	0.65	-4.62	9.18	0.04	11	0.947	0.574	1.94	3.34	0.58	-4.62	8.49	0.04	11	0.781	0.213	3.50	2.65	1.32	-1.69	8.69	0.29	11
Oscillospira	0.898	0.544	24.86	39.70	0.63	-52.96	102.68	0.04	11	0.982	0.900	4.94	38.38	0.13	-70.28	80.16	0.00	11	0.992	0.701	12.58	31.95	0.39	-50.05	75.21	0.04	11
f__Ruminococcaceae.g__*Ruminococcus*	0.854	0.207	-21.06	15.67	-1.34	-51.78	9.66	0.13	11	0.947	0.337	-15.44	15.34	-1.01	-45.52	14.63	0.12	11	0.992	0.637	-6.46	13.31	-0.49	-32.54	19.62	0.06	11
f__Ruminococcaceae.Other	0.973	0.914	3.43	31.11	0.11	-57.54	64.41	0.00	11	0.978	0.808	-7.34	29.44	-0.25	-65.05	50.37	0.01	11	0.992	0.634	11.99	24.49	0.49	-36.00	59.99	0.06	11
Megamonas	0.898	0.439	-9.26	11.53	-0.80	-31.85	13.34	0.06	11	0.947	0.455	-8.48	10.95	-0.77	-29.93	12.97	0.08	11	0.877	0.266	-10.43	8.90	-1.17	-27.86	7.01	0.26	11
Megasphaera	0.973	0.885	-2.08	14.10	-0.15	-29.71	25.55	0.00	11	0.978	0.729	-4.74	13.31	-0.36	-30.84	21.35	0.02	11	0.992	0.688	-4.59	11.14	-0.41	-26.44	17.25	0.05	11
Phascolarctobacterium	0.827	0.132	-49.82	30.54	-1.63	-109.68	10.03	0.17	11	0.947	0.228	-38.47	30.07	-1.28	-97.40	20.47	0.17	11	0.756	0.160	-37.10	24.64	-1.51	-85.39	11.19	0.35	11
Bilophila	0.898	0.399	-155.62	177.21	-0.88	-502.95	191.70	0.07	11	0.947	0.505	-117.31	170.13	-0.69	-450.76	216.14	0.06	11	0.992	0.851	-28.04	145.40	-0.19	-313.02	256.94	0.01	11
f__Enterobacteriaceae.g__	0.973	0.889	5.23	36.46	0.14	-66.23	76.69	0.00	11	0.982	0.935	2.87	34.61	0.08	-64.96	70.70	0.00	11	0.992	0.833	-6.27	28.99	-0.22	-63.10	50.56	0.01	11
Akkermansia	0.898	0.518	37.79	56.53	0.67	-73.02	148.59	0.04	11	0.978	0.722	19.91	54.47	0.37	-86.85	126.67	0.02	11	0.992	0.602	24.35	45.36	0.54	-64.56	113.27	0.07	11

	Startle									Escape Behavior								
	q value	*p* value	estimate	std error	*t*	CI 2.5%	CI 97.5%	r^2	df	*q* value	*p* value	estimate	std error	*t*	CI 2.5%	CI 97.5%	r^2	df
**o__Clostridiales.f__.g__**	0.650	**0.020***	11.67	4.38	2.67	3.09	20.26	0.38	13	0.451	0.068	10.15	5.26	1.93	-0.15	20.46	0.12	19
Sutterella	0.658	**0.039***	8.06	3.48	2.32	1.24	14.87	0.41	11	0.686	0.294	4.48	4.10	1.09	-3.56	12.52	0.06	14
Dialister	0.882	0.180	6.64	4.65	1.43	-2.47	15.74	0.27	11	0.143	**0.004***	12.41	3.60	3.45	5.36	19.46	0.28	13
**o__Clostridiales.Other.Other**	0.882	0.165	10.19	6.86	1.49	-3.26	23.64	0.25	11	0.451	**0.030***	15.44	6.47	2.39	2.76	28.11	0.19	15
**f__Erysipelotrichaceae.g__**	0.984	0.651	-4.42	9.51	-0.46	-23.07	14.22	0.05	10	0.451	**0.043***	17.87	7.69	2.32	2.79	32.95	0.20	10
Veillonella	0.920	0.446	-1.28	1.62	-0.79	-4.46	1.89	0.12	11	0.451	0.060	2.88	1.34	2.14	0.25	5.51	0.17	9
Bifidobacterium	0.984	0.938	-0.92	11.51	-0.08	-23.47	21.64	0.00	11	0.686	0.265	12.54	10.66	1.18	-8.34	33.43	0.08	11
Collinsella	0.920	0.383	-27.44	30.16	-0.91	-86.56	31.69	0.15	11	0.728	0.533	19.39	30.09	0.64	-39.58	78.37	0.03	11
Bacteroides	0.984	0.751	-0.16	0.49	-0.33	-1.13	0.81	0.02	10	0.686	0.370	-0.44	0.47	-0.94	-1.36	0.48	0.05	11
Parabacteroides	0.920	0.406	-3.80	4.39	-0.87	-12.40	4.80	0.14	11	0.686	0.169	-6.01	4.07	-1.47	-13.99	1.98	0.11	11
Prevotella	0.984	0.773	0.27	0.93	0.30	-1.55	2.09	0.02	10	0.855	0.723	0.33	0.91	0.36	-1.46	2.12	0.01	11
f__Rikenellaceae.g__	0.963	0.584	-12.12	21.44	-0.57	-54.14	29.89	0.07	11	0.686	0.350	-20.18	20.66	-0.98	-60.68	20.32	0.06	11
Streptococcus	0.984	0.881	0.76	4.95	0.15	-8.94	10.46	0.01	11	0.686	0.246	-5.62	4.58	-1.23	-14.60	3.36	0.08	11
Clostridium	0.920	0.421	-16.24	19.38	-0.84	-54.23	21.74	0.14	10	0.855	0.738	-6.72	19.55	-0.34	-45.03	31.60	0.01	11
f__Lachnospiraceae.g__	0.920	0.281	-1.61	1.42	-1.14	-4.39	1.17	0.21	11	0.820	0.646	0.69	1.45	0.47	-2.16	3.53	0.02	11
f__Lachnospiraceae.*g__.Ruminococcus.*	0.882	0.095	-28.82	15.72	-1.83	-59.62	1.98	0.37	11	0.855	0.760	5.49	17.54	0.31	-28.89	39.88	0.01	11
Blautia	0.984	0.797	-12.44	47.28	-0.26	-105.10	80.22	0.02	11	0.728	0.551	-28.19	45.83	-0.62	-118.01	61.63	0.02	11
Coprococcus	0.984	0.984	0.15	7.00	0.02	-13.57	13.87	0.00	11	0.686	0.451	-5.23	6.67	-0.78	-18.30	7.85	0.04	10
Dorea	0.984	0.979	-0.58	21.31	-0.03	-42.33	41.18	0.00	11	0.686	0.207	26.08	19.40	1.34	-11.93	64.10	0.10	11
Lachnospira	0.920	0.418	-1.93	2.29	-0.84	-6.41	2.55	0.14	11	0.686	0.409	1.92	2.23	0.86	-2.46	6.30	0.05	11
Roseburia	0.882	0.165	8.61	5.75	1.50	-2.65	19.88	0.30	10	0.912	0.902	0.78	6.24	0.13	-11.45	13.02	0.00	11
f__Lachnospiraceae.Other	0.920	0.345	-3.38	3.43	-0.99	-10.10	3.33	0.17	11	0.686	0.327	3.42	3.33	1.03	-3.11	9.95	0.06	11
f__Ruminococcaceae.g__	0.963	0.573	0.56	0.96	0.58	-1.32	2.43	0.07	10	0.686	0.458	-0.72	0.93	-0.77	-2.55	1.11	0.04	11
Faecalibacterium	0.882	0.187	1.49	1.05	1.41	-0.58	3.55	0.28	10	0.855	0.777	-0.33	1.13	-0.29	-2.54	1.89	0.01	11
Oscillospira	0.984	0.854	2.45	12.95	0.19	-22.93	27.82	0.01	11	0.728	0.527	8.17	12.48	0.65	-16.30	32.63	0.03	11
f__Ruminococcaceae.g__*Ruminococcus*	0.963	0.583	-3.02	5.34	-0.57	-13.48	7.44	0.07	11	0.686	0.372	-4.78	5.13	-0.93	-14.83	5.27	0.05	11
f__Ruminococcaceae.Other	0.984	0.861	-1.79	9.96	-0.18	-21.31	17.73	0.01	11	0.912	0.912	-1.12	9.81	-0.11	-20.35	18.12	0.00	11
Megamonas	0.984	0.735	-1.31	3.77	-0.35	-8.71	6.08	0.03	11	0.686	0.252	-4.25	3.51	-1.21	-11.12	2.63	0.08	11
Megasphaera	0.963	0.509	-3.03	4.43	-0.68	-11.71	5.66	0.10	11	0.882	0.828	-0.99	4.44	-0.22	-9.68	7.71	0.00	11
Phascolarctobacterium	0.920	0.377	-9.66	10.48	-0.92	-30.21	10.88	0.16	11	0.686	0.418	-8.73	10.37	-0.84	-29.07	11.60	0.04	11
Bilophila	0.984	0.778	-16.88	58.46	-0.29	-131.46	97.69	0.02	11	0.686	0.449	-44.30	56.34	-0.79	-154.73	66.13	0.04	11
f__Enterobacteriaceae.g__	0.920	0.411	-9.72	11.35	-0.86	-31.96	12.52	0.14	11	0.686	0.429	9.17	11.14	0.82	-12.66	30.99	0.04	11
Akkermansia	0.984	0.905	-2.28	18.54	-0.12	-38.62	34.07	0.00	11	0.686	0.261	20.30	17.07	1.19	-13.15	53.75	0.08	10

Linear mixed effect model with t-test, FDR multiple comparison correction, bold formatting with * denotes $p < 0.05$, n = 14. Source data for this table are provided as a Source Data file.

Supplementary Table 3. Genera at 1 month of age associated with brain volumes at 1 month of age

	1-Month Hippocampus									1-Month Amygdala									1-Month mPFC								
	q value	*p* value	estimate	std error	*t*	CI 2.5%	CI 97.5%	r^2	df	*q* value	*p* value	estimate	std error	*t*	CI 2.5%	CI 97.5%	r^2	df	*q* value	*p* value	estimate	std error	*t*	CI 2.5%	CI 97.5%	r^2	df
Streptococcus**	0.132	**0.009**	-257	90	-2.9	-443	-71	0.26	23	**0.021****	**0.001**	-355	98	-3.6	-558	-152	0.36	23	0.123	**0.012**	-7612	2778	-2.7	-13360	-1865	0.24	23
Bacteroides	0.795	0.211	102	79	1.3	-62	265	0.06	23	0.074	**0.010**	233	83	2.8	62	404	0.23	23	0.628	0.281	2689	2436	1.1	-2351	7729	0.04	23
Staphylococcus	0.879	0.293	-3963	3683	-1.1	-11582	3655	0.05	23	0.246	**0.049**	-8456	4070	-2.1	-16875	-37	0.15	23	0.156	**0.031**	-239046	104203	-2.3	-454607	-23485	0.18	23
**f__Lachnospiraceae. Other**	0.942	0.752	76	238	0.3	-416	568	0.00	23	0.778	0.531	177	278	0.6	-398	751	0.02	23	0.123	**0.016**	16622	6417	2.6	3347	29897	0.21	23
Bifidobacterium	0.942	0.514	-109	164	-0.7	-449	231	0.02	23	0.645	0.299	-202	190	-1.1	-596	192	0.04	23	0.628	0.247	-5859	4930	-1.2	-16058	4339	0.05	23
Enterococcus	0.268	0.358	5553	2489	2.2	403	10702	0.16	23	0.778	0.460	2395	3187	0.8	-4199	8988	0.02	23	0.999	0.945	5829	84011	0.1	-168000	179619	0.00	23
Lactobacillus	0.952	0.952	17	276	0.1	-555	588	0.00	23	0.933	0.809	79	324	0.2	-591	750	0.00	23	0.905	0.543	5176	8386	0.6	-12171	22522	0.01	23
f__Clostridiaceae.g__	0.942	0.879	-38	248	-0.2	-550	474	0.00	23	0.778	0.622	-145	290	-0.5	-744	454	0.01	23	0.969	0.775	-2191	7569	-0.3	-17847	13466	0.00	23
Clostridium	0.942	0.588	66	120	0.6	-181	313	0.01	23	0.645	0.301	146	138	1.1	-139	431	0.04	23	0.628	0.293	3863	3590	1.1	-3564	11290	0.04	23
f__Clostridiaceae.Other	0.942	0.854	-101	544	-0.2	-1226	1024	0.00	23	0.778	0.601	-338	636	-0.5	-1653	978	0.01	23	0.920	0.624	-8234	16569	-0.5	-42510	26043	0.01	23
f__Lachnospiraceae.g__	0.795	0.212	1382	1077	1.3	-847	3611	0.07	23	0.645	0.241	1530	1271	1.2	-1100	4159	0.06	23	0.999	0.999	-23	34133	0.0	-70633	70588	0.00	23
f__Lachnospiraceae.g__. *Ruminococcus*.	0.942	0.527	1867	2904	0.6	-4140	7874	0.02	23	0.778	0.495	2364	3407	0.7	-4685	9412	0.02	23	0.784	0.418	72868	88363	0.8	-110000	255659	0.03	23
Veillonella	0.942	0.725	-49	138	-0.4	-333	236	0.00	23	0.956	0.953	10	162	0.1	-326	345	0.00	23	0.920	0.675	1788	4203	0.4	-6907	10483	0.01	23
f__Enterobacteriaceae.g_	0.942	0.519	-172	263	-0.7	-717	372	0.02	23	0.349	0.093	-514	293	-1.8	-1121	93	0.11	23	0.628	0.184	-10701	7820	-1.4	-26878	5477	0.07	23
o__Pseudomonadales. Other.Other	0.942	0.843	324	1620	0.2	-3027	3674	0.00	23	0.956	0.956	107	1905	0.1	-3833	4047	0.00	23	0.999	0.907	5882	49595	0.1	-96713	108476	0.00	23

Linear regression with t-test, FDR multiple comparison correction, ** denotes significance after FDR correction, bold formatting denotes $p < 0.05$, n = 27, covariates: age at 1 month scan, sex. Source data for this table are provided as a Source Data file.

Supplementary Table 4. Genera at 1 month of age associated with brain volumes at 1 year of age

	1-Year Hippocampus									1-Year Amygdala									1-Year mPFC								
	q value	*p* value	estimate	std error	*t*	CI 2.5%	CI 97.5%	r^2	df	*q* value	*p* value	estimate	std error	*t*	CI 2.5%	CI 97.5%	r^2	df	*q* value	*p* value	estimate	std error	*t*	CI 2.5%	CI 97.5%	r^2	df
Bacteroides	0.694	0.432	252	307	0.8	-433	937	0.06	10	0.992	0.873	26	160	0.2	-330	383	0.00	10	0.262	**0.035**	-18833	7722	-2.4	-36038	-1628	0.36	10
**f__Lachnospiraceae.Other**	0.645	0.137	896	553	1.6	-337	2129	0.18	10	0.298	**0.031**	616	246	2.5	69	1164	0.33	10	0.491	0.131	27842	16940	1.6	-9902	65585	0.18	10
Veillonella	0.645	0.262	789	663	1.2	-689	2266	0.10	10	0.302	0.060	629	297	2.1	-33	1290	0.27	10	0.052	**0.003**	52921	13903	3.8	21943	83899	0.54	10
**f__Enterobacteriaceae.g__**	0.879	0.820	184	792	0.2	-1579	1948	0.00	10	0.298	**0.040**	-758	321	-2.4	-1472	-43	0.31	10	0.975	0.325	-23990	23164	-1.0	-75604	27623	0.08	10
Bifidobacterium	0.694	0.548	630	1013	0.6	-1627	2887	0.05	10	0.992	0.731	183	517	0.4	-970	1336	0.02	10	0.294	0.059	56018	26288	2.1	-2556	114592	0.39	10
Staphylococcus	0.913	0.913	-1129	10100	-0.1	-23634	21376	0.00	10	0.992	0.949	-335	5097	-0.1	-11692	11021	0.00	10	0.983	0.650	-143738	307028	-0.5	-827839	540363	0.02	10
Enterococcus	0.645	0.211	-8713	6526	-1.3	-23254	5828	0.13	10	0.992	0.511	2380	3493	0.7	-5403	10163	0.04	10	0.983	0.698	86115	215836	0.4	-394796	567027	0.01	10
Lactobacillus	0.645	0.300	-710	650	-1.1	-2158	737	0.08	10	0.992	0.433	-274	336	-0.8	-1022	474	0.05	10	0.983	0.823	4829	21055	0.2	-42085	51742	0.00	10
Streptococcus	0.792	0.686	-130	313	-0.4	-828	567	0.02	10	0.992	0.866	-27	159	-0.2	-382	327	0.00	10	0.983	0.852	-1853	9678	-0.2	-23417	19711	0.00	10
f__Clostridiaceae.g__	0.694	0.555	-436	714	-0.6	-2026	1154	0.03	10	0.992	0.724	-132	364	-0.4	-944	679	0.01	10	0.983	0.488	-15666	21764	-0.7	-64159	32827	0.04	10
Clostridium	0.645	0.078	-955	486	-2.0	-2039	129	0.22	10	0.780	0.208	-358	266	-1.4	-950	234	0.12	10	0.983	0.825	-3982	17541	-0.2	-43065	35102	0.00	10
f__Clostridiaceae.Other	0.694	0.540	-1264	1991	-0.6	-5702	3173	0.03	10	0.992	0.674	-439	1015	-0.4	-2701	1822	0.01	10	0.989	0.989	-869	62376	0.0	-139851	138114	0.00	10
f__Lachnospiraceae.g__	0.645	0.092	301271	162000	1.9	-59392	662000	0.22	10	0.992	0.992	-988	94742	0.0	-212087	210111	0.00	10	0.983	0.814	-1392040	5751246	-0.2	-14206613	11422534	0.00	10
f__Lachnospiraceae.g__ .*Ruminococcus*.	0.645	0.301	-7591	6955	-1.1	-23088	7906	0.08	10	0.992	0.443	-2877	3598	-0.8	-10894	5140	0.05	10	0.983	0.844	45592	225499	0.2	-456852	548035	0.00	10
o__Pseudomonadales .Other.Other	0.694	0.425	-8147	9786	-0.8	-29951	13658	0.06	10	0.992	0.729	-1806	5072	-0.4	-13109	9496	0.01	10	0.989	0.928	28586	310639	0.1	-663562	720733	0.00	10

Linear regression with t-test, FDR multiple comparison correction, bold formatting denotes $p < 0.05$, n = 14, covariates: age at 1 year scan, sex. Source data for this table are provided as a Source Data file.

Supplementary Table 5. Sequencing primers

Primer Name	Primer Sequence
8F	5’ TCGTCGGCAGCGTCAGATGTGTATAAGAGACAGAGAGTTTGATCCTGGCTCAG3’
BifidoF	5’ TCGTCGGCAGCGTCAGATGTGTATAAGAGACAGAGGGTTCGATTCTGGCTCAG3’
338R	5’ GTCTCGTGGGCTCGGAGATGTGTATAAGAGACAGGCTGCCTCCCGTAGGAGT3’

Supplementary Table 6. Description of demographic, medical, and feeding variables assessed

Variable	Method	Details
income	parental report	participants were stratified into low (<200% federal poverty level), middle (200%-400% federal poverty level), high (>400% federal poverty level) based on the reported household income and federal poverty level for the household size at the year of visit)
maternal ethnicity	parental report	
paternal ethnicity	parental report	
maternal age at birth	parental report & medical record review	
paternal age at birth	parental report	
maternal education	parental report	
paternal education	parental report	
material psychiatric history	parental report or medical record	binary variable created for self-report or medical record positive for psychiatric history of schizophrenia spectrum disorder, bipolar disorder, depressive disorder, anxiety disorder, obsessive-compulsive disorder, attention-deficit hyperactivity disorder, Tourette's syndrome, or autism spectrum disorders
maternal infection during pregnancy	parental report	
maternal pre-pregnancy BMI	parental report or medical record	
maternal state anxiety at 1 year	State-Trait Anxiety Inventory	

maternal trait anxiety at 1 year	State-Trait Anxiety Inventory	
gestational age at birth	medical record	
birth weight	medical record	
sex	medical record	
having older siblings	medical record	
breastfeeding at 1 year	parental report	
receiving formula at 1 year	parental report	
receiving formula at 6 months	parental report	
vitamin D supplementation at 1 month	parental report	at least 3 days/week for 2 weeks
vitamin D supplementation at 6 months	parental report	at least 3 days/week for 2 weeks
age at first feeding of food other than breastmilk or formula	parental report	
cereals at 6 months	parental report	
milks at 1 year	parental report	
french fries at 1 year	parental report	
sweet food or drinks at 1 year	parental report	
nuts at 1 year	parental report	
daycare attendance during 1st year of life	parental report	
fever in previous 2 weeks	parental report	
antibiotics during 1st year of life	parental report & medical record review	
age in days at visit 1		

age in days at visit 2		
negative life events	Life Experiences Survey at 12 months	
positive life events	Life Experiences Survey at 12 months	
total life events	Life Experiences Survey at 12 months	
research assistant for behavioral assessment		two different research assistants performed the stranger or mask task role during behavioral assessments

Supplementary Table 7. Mask Task coding criteria

Intensity of Facial Fear	0-3	0 – No response<br>1 – only one facial region shows codable movement, expression is different from baseline but ambiguous<br>2 – 2 facial regions show codable movement, or expression in one region is very clear – not an extreme fear reaction<br>3 – facial fear reaction with all 3 facial regions (brows, eyes, mouth) showing strong facial fear
Intensity of Vocal Distress	0-3	0 – No response<br>1 – Mild vocalization that may be difficult to identify, whimpering, limited short duration<br>2 – Longer low intensity cry to non-muted crying<br>3 – Full intensity cry/scream
Intensity of Bodily Fear	0-3	0 – No response<br>1 – Decreased activity: sudden decrease from baseline of previous episode, will be coded often – probably not meaningful for measuring fear/anxiety<br>2 – Tensing: muscles tense, along with decreased movement<br>3 – Freezing/trembling: more pronounced than 2, often involves entire body, trembling from extreme tension
Intensity of Escape Behavior	0-3	0 – no behavior<br>1 – Mild: turning away or sinking in chair (in combo with distress, not turning around smiling at mom)<br>2 – Moderate escape: full body movements, arching back<br>3 – Vigorous escape: linked, continuous full body movements, arching back, attempts to escape
Startle Response	0=no<br>1=yes	1 = sudden large change in bodily position, facial expression, vocalization. Should occur immediately after initial presentation of mask
Parent Behavior	0-2	0 – not interfering<br>1 – mild interference, some comments to infant, not emotionally loaded<br>2 – interfering, emotionally loaded statements, soothing
