# Supplementary material for: Infant gut microbiome composition is associated with non-social fear behavior in a pilot study
Source: Nat Commun. 2021 Jun 2;12:3294. doi: 10.1038/s41467-021-23281-y (PMC8172562; doi:10.1038/s41467-021-23281-y)
Supplement: Supplementary file 3 — Reporting Summary [file 41467_2021_23281_MOESM3_ESM.pdf]

nature research

Corresponding author(s): Rebecca Knickmeyer

Last updated by author(s): Oct 14, 2020

Reporting Summary

Nature Research wishes to improve the reproducibility of the work that we publish. This form provides structure for consistency and transparency in reporting. For further information on Nature Research policies, see Authors & Referees and the Editorial Policy Checklist.

Statistics

For all statistical analyses, confirm that the following items are present in the figure legend, table legend, main text, or Methods section.

n/a	Confirmed	
☐	☒	The exact sample size (n) for each experimental group/condition, given as a discrete number and unit of measurement
☐	☒	A statement on whether measurements were taken from distinct samples or whether the same sample was measured repeatedly
☐	☒	The statistical test(s) used AND whether they are one- or two-sided<br>*Only common tests should be described solely by name; describe more complex techniques in the Methods section.*
☐	☒	A description of all covariates tested
☐	☒	A description of any assumptions or corrections, such as tests of normality and adjustment for multiple comparisons
☐	☒	A full description of the statistical parameters including central tendency (e.g. means) or other basic estimates (e.g. regression coefficient) AND variation (e.g. standard deviation) or associated estimates of uncertainty (e.g. confidence intervals)
☐	☒	For null hypothesis testing, the test statistic (e.g. F, t, r) with confidence intervals, effect sizes, degrees of freedom and P value noted<br>*Give P values as exact values whenever suitable.*
☒	☐	For Bayesian analysis, information on the choice of priors and Markov chain Monte Carlo settings
☒	☐	For hierarchical and complex designs, identification of the appropriate level for tests and full reporting of outcomes
☐	☒	Estimates of effect sizes (e.g. Cohen's d, Pearson's r), indicating how they were calculated

Our web collection on statistics for biologists contains articles on many of the points above.

Software and code

Policy information about availability of computer code

Data collection	No software was used to collect data
Data analysis	QIIME 1, PICRUSt 1.1.0, R 3.5.1, AutoSeg 3.3.2, NeoSeg 1.0.8, ITK-SNAP 3.4, ChimeraSlayer, FastQC 0.11.8, configureBclToFastq

For manuscripts utilizing custom algorithms or software that are central to the research but not yet described in published literature, software must be made available to editors/reviewers. We strongly encourage code deposition in a community repository (e.g. GitHub). See the Nature Research guidelines for submitting code & software for further information.

Data

Policy information about availability of data

All manuscripts must include a data availability statement. This statement should provide the following information, where applicable:

- Accession codes, unique identifiers, or web links for publicly available datasets
- A list of figures that have associated raw data
- A description of any restrictions on data availability

16S rRNA amplicon sequencing data is available through the NCBI repository under accession PRJNA547558. The KEGG database used in PICRUSt analyses is accessible at https://www.genome.jp/kegg/. Data that support the findings of this study are included as source data or are available from the corresponding author on reasonable request.

Field-specific reporting

Please select the one below that is the best fit for your research. If you are not sure, read the appropriate sections before making your selection.

☐ Life sciences ☒ Behavioural & social sciences ☐ Ecological, evolutionary & environmental sciences

For a reference copy of the document with all sections, see nature.com/documents/nr-reporting-summary-flat.pdf

Behavioural & social sciences study design

All studies must disclose on these points even when the disclosure is negative.

Study description

This was a prospective longitudinal cohort study of 34 infants followed from 1 month to 1 year of age.

Research sample

This research sample was comprised of 34 infants recruited from central North Carolina hospitals after birth. To reduce confounding effects of birth mode, antibiotic exposure, and feeding practices, we had stringent inclusion/exclusion criteria. Inclusion criteria for participation in the study were vaginal delivery and exclusive breastfeeding until the first study visit at 1 month. Participants were excluded for maternal antibiotic usage two weeks before delivery (including Group B Streptococcal prophylaxis), antibiotics given to the infant before the first study visit, neonatal intensive care unit stay, birth weight <2500 grams, gestational age <37 weeks, major maternal medical illness, prenatal drug use, primary language other than English, and fetal ultrasound abnormalities. Prenatal, labor and delivery, and pediatric medical records were reviewed to ensure that participants met study inclusion/exclusion criteria. Participants had two study visits at UNC (median age at visit 1 = 30 days, median age at visit 2 = 384 days) and one phone interview at 6 months. The cohort was representative of North Carolina in terms of race and ethnicity. Brief cohort demographic information is as follows: 68% male, maternal ethnicity of 77% white and 24% black, family income of 38% high, 38% middle, and 24% low income. See methods for further detail.

Sampling strategy

Convenience sampling was used to recruit participants for this study. Infants meeting inclusion criteria were sent information about the study by mail and email. Interested participants were then screened with exclusion criteria as listed above. This sample was from a pilot feasibility study that had a small sample size by design. Sample size is similar to other infant microbiome studies: DOI: 10.1038/s41467-018-07631-x (n = 33) , DOI: 10.1111/cea.12253 (n = 47).

Data collection

Observational Behavioral Data: Strange Situation and Mask Task Lab-TAB behavioral paradigms were used to assess social and non-social fear, respectively. Paradigms were video recorded with 6 recessed video cameras for later coding. All videos for Strange Situation and Mask Task were coded by one person who was reliable with a second coder with >0.8 ICC. Both coders were blinded to microbiome predictors. See methods for further detail.

Parent Report: Before the visit at 1 year of age, mothers were mailed a copy of the Infant Behavior Questionnaire – Revised (IBQ-R) to complete and bring to the study visit. Questionnaires were scored to generate a composite fear score to use as a parent reported infant fear behavior outcome at age 1 year.

Setting: 3 research coordinators, the mom and baby were present during the behavioral testing session. No one else was present during active behavioral data collection.

Timing

Participants were recruited and had study visits from January 2014 to November 2015

Data exclusions

Subjects were excluded from the dataset if found to not meet inclusion/exclusion criteria after medical record review. One subject at 1 month was excluded after sequencing for not meeting study criteria after medical record review. One sample was excluded at 1 month and at 1 year for sequencing reads <.1% of total.

No strange situation or IBQ-R data warranted exclusion.

For the mask task paradigm, if the infant became upset by placement in the high chair before the start of the mask task trial, the paradigm was not started as it would confound interpretation (n = 10).

These exclusion criteria were pre-established.

Non-participation

659 families were sent study information after initially meeting study criteria. Of those, 176 did not subsequently meet study criteria after screening. 212 were not interested in participating. 237 did not respond to study recruitment materials. 34 of subjects were enrolled in the study.

Randomization

Participants were not allocated to groups in this study.

Reporting for specific materials, systems and methods

We require information from authors about some types of materials, experimental systems and methods used in many studies. Here, indicate whether each material, system or method listed is relevant to your study. If you are not sure if a list item applies to your research, read the appropriate section before selecting a response.

Materials & experimental systems

n/a	Involved in the study
☒	☐ Antibodies
☒	☐ Eukaryotic cell lines
☒	☐ Palaeontology
☒	☐ Animals and other organisms
☐	☒ Human research participants
☒	☐ Clinical data

Methods

n/a	Involved in the study
☒	☐ ChIP-seq
☒	☐ Flow cytometry
☐	☒ MRI-based neuroimaging

Human research participants

Policy information about studies involving human research participants

Population characteristics: Please see above

Recruitment: Please see above. As this was a prospective longitudinal cohort study, there is a possibility for self-selection bias. However our final cohort represented the diversity of North Carolina in terms of maternal ethnicity, and demonstrated variations in feeding practices across the first year of life. We do not expect self-selection bias to impact these findings.

Ethics oversight: This study was approved by the Institutional Review Board of the University of North Carolina at Chapel Hill.

Note that full information on the approval of the study protocol must also be provided in the manuscript.

Magnetic resonance imaging

Experimental design

Design type: n/a

Design specifications: n/a

Behavioral performance measures: n/a

Acquisition

Imaging type(s): Structural

Field strength: 3T

Sequence & imaging parameters: TIM-Trio scanner (Siemens Medical System, Erlangen, Germany)

1 Month:
MP RAGE (repetition time = 1900 ms, echo time = 3.89 ms, 7 degree flip angle, 0.8x0.8x0.8 mm voxel resolution), T2 (turbo-spin echo sequence, repetition time = 3200 ms, echo time = 406-410 ms, 120 degree flip angle, 0.8x0.8x0.8 mm voxel resolution).

1 Year:
MP RAGE (repetition time = 1900 ms, echo time = 3.1 ms, 7 degree flip angle, 0.8x0.8x0.8 mm voxel resolution).

Area of acquisition: Whole brain scan; analysis limited to hippocampi, amygdalae, and medial prefrontal cortex grey matter volumes

Diffusion MRI: ☐ Used ☒ Not used

Preprocessing

Preprocessing software: inhomogeneity correction via N4 as implemented in AutoSeg v3.3.2

Normalization: rigid registration into MNI pediatric space via BRAINSFit as implemented in AutoSeg v3.3.2

Normalization template: MNI 1-2 year pediatric template

Noise and artifact removal: None (this is structural T1weighted data, not fMRI)

Volume censoring: N/A

Statistical modeling & inference

Model type and settings	n/a
Effect(s) tested	n/a

Specify type of analysis: ☐ Whole brain ☐ ROI-based ☐ Both

Statistic type for inference (See Eklund et al. 2016)	n/a
Correction	n/a

Models & analysis

n/a	Involved in the study
☒	☐ Functional and/or effective connectivity
☒	☐ Graph analysis
☒	☐ Multivariate modeling or predictive analysis
